# *EML4-ALK* rearrangement in primary malignant fibrous histiocytoma of the lung treated with alectinib: A case report

**DOI:** 10.3389/fonc.2022.978327

**Published:** 2022-09-05

**Authors:** Shuai Zhang, Xuqiang Liao, Jiawei Chen

**Affiliations:** ^1^ Department of Radiation Oncology, Hainan General Hospital, Hainan Affiliated Hospital of Hainan Medical University, Haikou, China; ^2^ Department of Thoracic Surgery, Hainan General Hospital, Hainan Affiliated Hospital of Hainan Medical University, Haikou, China

**Keywords:** treatment, malignant fibrous histiocytoma (MFH), alectinib, lung, case report

## Abstract

Primary malignant fibrous histiocytoma of the lung (PMFHL) is extremely rare. It is more common in the right lung and has no specific symptoms. Lymph node metastasis is rare, but hematogenous metastasis is more common. The common metastatic sites are the brain and bone. In this study, a 59-year-old male patient was diagnosed with PMFHL with brain metastasis due to persistent cough and blood in the sputum for the past week. Genetic testing revealed *EML4-ALK* gene rearrangement (fusion). We first used alectinib in a patient with advanced PMFHL with *EML4-ALK* gene rearrangement (fusion) accompanied by brain metastasis. The treatment was effective and successfully delayed the development of the disease. Satisfactory results were observed, with an overall survival time of 19 months. Therefore, genetic testing in PMFHL and the choice of treatment plan are important. Local treatment methods, including surgery and radiotherapy, are important when the disease is less advanced. Multidisciplinary discussion is recommended for the best prognosis.

## Introduction

Malignant fibrous histiocytoma (MFH) is the most common malignant mesenchymal tumor in adults. MFH can occur in a variety of organs, especially the trunk part of the extremities and the deep musculature of the retroperitoneum, but it rarely originates in the lung and more often metastasizes to the lung from other organs (1). Surgery is the main treatment for primary MFH of the lung (PMFHL). The effectiveness of adjuvant radiotherapy after surgery is unclear.

The pathology of PMFHL is divided into the fibroblast type, histiocytic type, and inflammatory cell type. The postoperative gross specimens are mostly large tumors with a diameter of >5 cm, with pseudocapsules and gray–white sections with necrosis in the center. Fibrillar cells and collagen fibers are arranged in bundles of spokes, and histiocytic cells with large variation, mononuclear and multinucleated giant cells, and foam cells with nuclear atypia are observed under light microscopy. The immunohistochemical characteristics are as follows: α1-antitrypsin and α1-antichymotrypsin, vimentin (+), S-100 protein (-), and myosin (-) (2). Because the growth of PMFHL is a centrifugal spherical enlargement phenomenon, the surrounding normal tissue is compressed and changed in layers, forming a relatively tight “compression zone”, and a tissue reaction phenomenon occurs around the “compression zone” to form a pseudocapsule (3).

Because of its complex and heterogeneous pathological features, the lack of specific immunohistochemical markers, and no lineage specificity, the clinical diagnosis of PMFHL is controversial. We strongly recommend that core needle biopsy is required for pathological tissue. In this paper, we report a rare case of PMFHL with brain metastasis and EML4-ALK gene rearrangement (fusion). We investigated the clinical diagnosis, treatment characteristics, and prognosis of this disease and analyzed the application value and challenges of genetic testing and targeted drugs over the whole course of treatment.

## Case description

All procedures performed in human participants followed the ethical standards of the institutional and/or national research committee(s) and with the Helsinki Declaration (as revised in 2013). Written informed consent was obtained from the patient.

**Figure 1:**
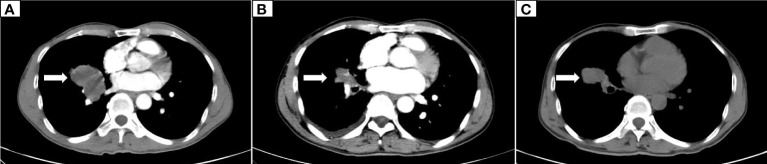
Dynamic changes on chest CT. **(A)** The initial CT scan on January 23, 2019, showed an irregular soft tissue mass measuring 5.7 cm × 4.5 cm in the middle and lower lobes of the right lung. **(B)** A CT scan on December 9, 2019, showed that the right lung lesion was reduced to 2.5 cm × 1.5 cm. **(C)** A CT scan on May 5, 2020, showed progression of the right lung lesion, which had grown to 4.5 cm × 3.5 cm.

**Figure 2:**
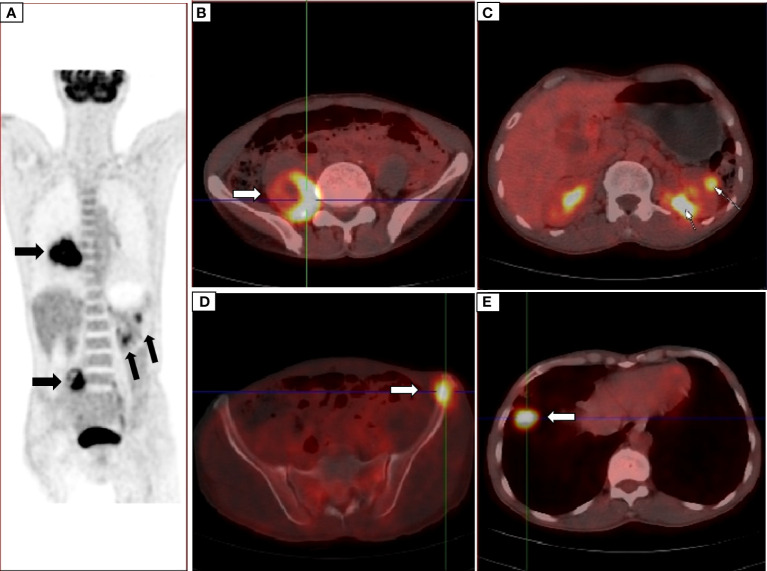
PET/CT showed nodules in the right middle and lower lobes near the hilar area **(A)** the right psoas muscle area **(B)** and the gastrosplenic space and multiple nodules in the left renal parenchyma **(C)** uneven density of the left iliac bone **(D)** multiple round nodules in the right lung **(E)** and increased FDG metabolism, suggesting malignant lesions.

**Figure 3:**
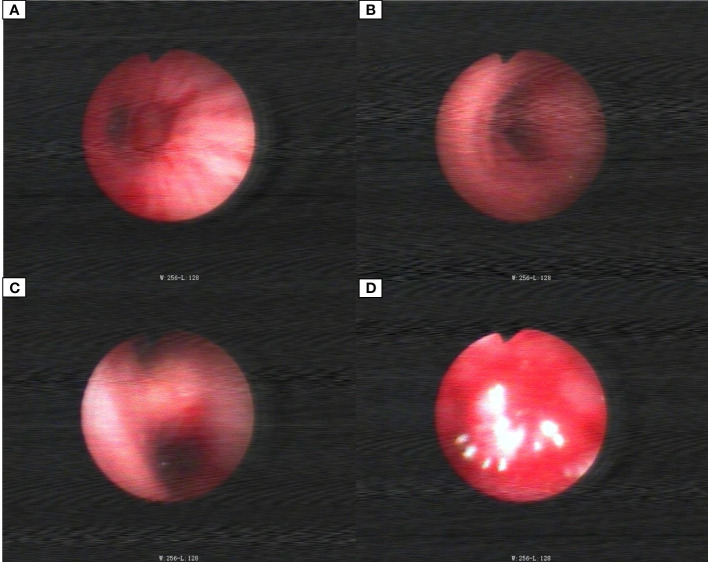
Bronchoscopic images. **(A)** Middle bronchial tubes. **(B)** Right main stem bronchi. **(C)** Right middle lobe bronchial. **(D)** Right lower lobe bronchial tube.

**Figure 4:**
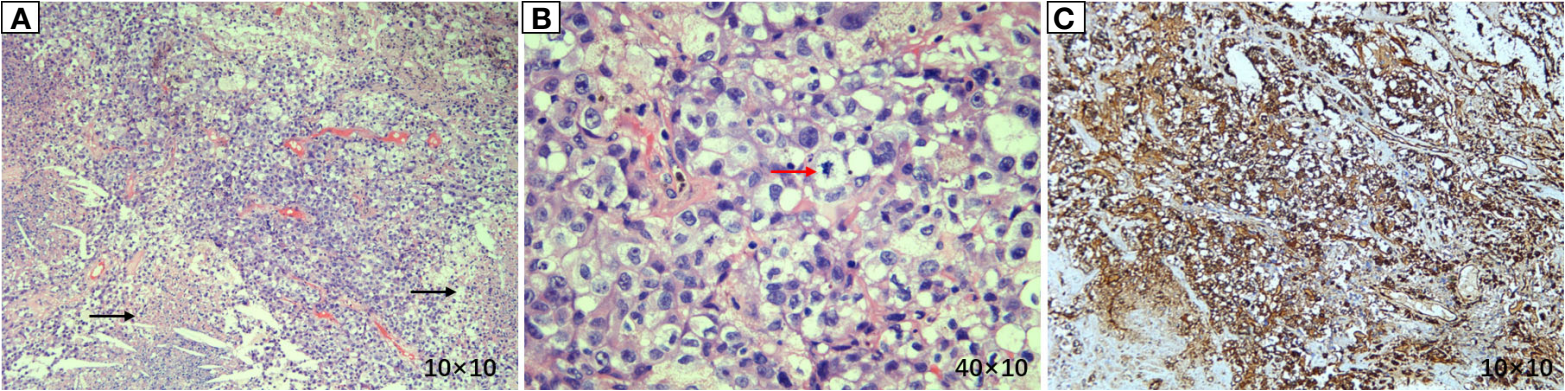
Pathological results of lung lesion biopsy. **(A)** (Hematoxylin and eosin (HE) × 100) Atypical tumor cells were arranged in a sheet-like manner, with vascular proliferation and necrosis. **(B)** (HE × 400) Atypical tumor cells showed large nuclei, hyperchromatic nuclei, prominent nucleoli, two or more nucleoli, eosinophilic or translucent cytoplasm, and pathological mitosis. **(C)** (IHC × 100) Vimentin was diffusely expressed, suggesting tumors derived from mesenchymal tissues.

**Figure 5:**
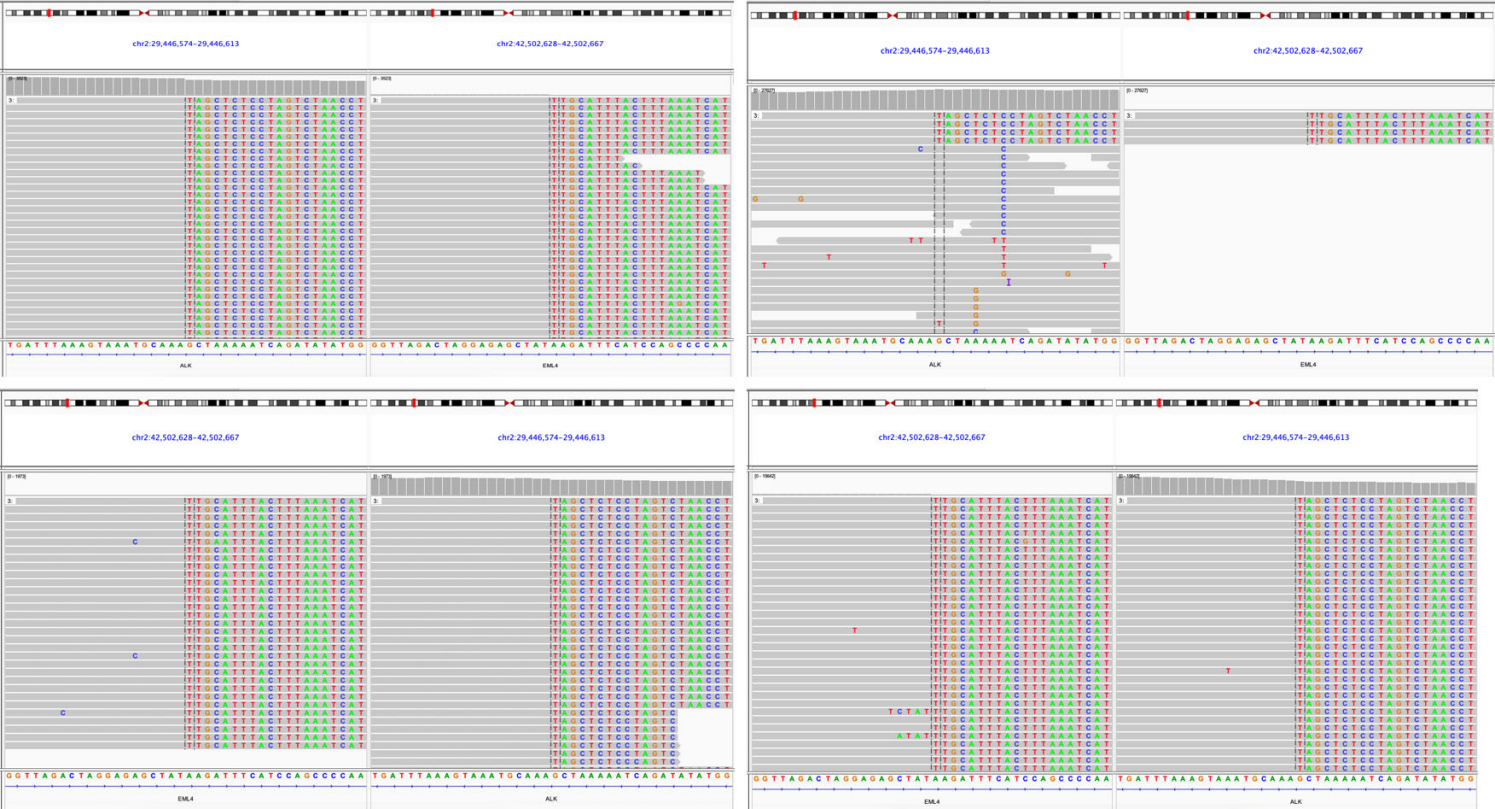
Genomic map derived from the biopsied tissue samples of four lung lesions.

The patient, a 59-year-old male, was admitted to the Department of Thoracic Surgery on January 22, 2019, with coughing with no obvious cause, white sticky sputum with occasional bloody sputum, chest tightness but no chest pain, hemoptysis, shortness of breath, headache, and dizziness. The patient had a history of smoking for more than 30 years (1-2 packs/day). Chest computed tomography (CT) showed irregular soft tissue nodules and mass shadows in the right middle and lower lobes near the hilar area and the anterior basal segment of the right lower lobe. The larger nodule was located next to the right hilum, with a larger cross-section of approximately 5.7 cm × 4.5 cm and a lobular shape. The boundary between the lesion and the right hilum was unclear, straddling the oblique fissure pleura, with uneven enhancement on enhanced scanning, and bronchial stenosis in the middle and lower lobes of the right lung was narrow. The right hilar lymph nodes were enlarged, and the mediastinal lymph nodes were slightly enlarged (**Figure 1**). Positron-emission tomography (PET/CT) results showed the following: 1. increased ^18^F-fluorodeoxyglucose (FDG) metabolism in nodules and masses at the right middle and lower lobe near the hilar area with a maximum standardized uptake value (SUV max) of 13.1, the right psoas muscle area, the gastrosplenic space, and the left inferior abdominal wall; multiple round-like nodules in the right lung with elevated FDG metabolism; a left parietal nodular lesion surrounded by flaky edema and increased FDG metabolism; uneven bone density in the left iliac bone with increased FDG metabolism; and multiple nodules in the left renal parenchyma with increased FDG metabolism, all suggesting malignant tumor lesions; and 2. slightly larger bilateral hilar and mediastinal lymph nodes, increased FDG metabolism, and the possibility of metastasis. Follow-up examination was recommended (**Figure 2**). Fiberoptic bronchoscopy examination showed that in the right bronchus, the right middle bronchial mucosa was swollen and bulged, cauliflower-like masses grew in the right middle lobe and the lower lobe segment of the bronchial lumen, the lumen was completely occluded, and the lesion was greater than 2 cm from the protuberance (**Figure 3**). Pathology of the specimen under bronchoscopy showed a malignant tumor (middle right, lower right). After immunohistochemical labeling, a malignant soft tissue tumor was reported (middle right, lower right), which was consistent with MFH. The nucleus of the tumor cells was irregularly shaped, mainly shuttle-shaped, large nucleated cells were observed, and nuclear divisions were easily observed and diffusely distributed. Immunohistochemical labeling showed vimentin (+), α1-antitrypsin (+), α1-antichymotrypsin (+), CK19 (some +), S-100 (-), desmin (-), MyoD1 (-), HHF35 (-), Fli-1 (-), ALK (-), CK7 (-), myogenin (-), SMA (-), CD34 (-), CD56 (-), Bcl-2 (-), CD99 (partial +), Ki-67>60%, CK (-), TFF-1 (-), napsin A (-), P63 (-), P40 (-), CK7 (-), and CK20 (-). See **Figure 4** for details. After a multidisciplinary discussion, the patient was considered to have MFH with multiple systemic metastases, without surgical indications and with poor chemotherapy efficacy. We recommended genetic testing to evaluate whether targeted immunotherapy was appropriate. The results of genetic testing showed that the ALK gene had the *EML4-ALK* (E6:A20) gene rearrangement (fusion) with an abundance of 8.93% (**Figure 5**). The tumor mutation burden (TMB) was 1.6 mutations/Mb. Microsatellite instability results showed microsatellite stability. Programmed death-ligand 1 (PD-L1) was detected using immunohistochemistry (clone number 22C3). The tumor proportion score (TPS) was 80%. Considering the possibility of *ALK* gene mutation, the efficacy of first-line alectinib was better than that of crizotinib, and the immunotherapy effect was poor. He was started on alectinib 600 mg orally twice daily. The symptoms completely disappeared after 1 month of administration.

**Figure 6:**
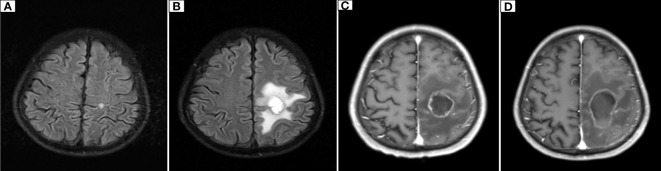
Dynamic changes on cranial MR. **(A)** The initial cranial MR on January 28, 2019, showed a nodular lesion in the left parietal lobe with a diameter of approximately 0.6 cm and a clear margin. **(B)** On December 6, 2019, cranial MR showed that the left parietal lobe nodule was enlarged to 2.1 cm × 2.3 cm, with blurred edges and patchy surrounding edema. **(C)** On March 23, 2020, cranial MR showed that the left parietal lobe nodule had grown to 3.0 cm × 2.3 cm and had blurred edges and visible patchy edematous lesions around it. **(D)** Cranial MR showed that the left parietal nodular lesion had grown to 4.5 cm×2.5 cm, with blurred edges and patchy edematous lesions.

The patient was hospitalized on December 3, 2019, due to right limb weakness with numbness. Cranial magnetic resonance (MR) showed that the nodular-shaped abnormal signal of the left parietal lobe was significantly larger than before, with an increase in the small amount of edema in the surrounding area. The tumor was considered to be metastatic (**Figure 6**). Because the patient had stable pulmonary lesions and progressing craniocerebral lesions, neurosurgeons considered resection of the left parietal lobe metastases, and the patient was informed of the above conditions. The patient did not consent to surgical resection due to the risk of surgery. Stereotactic radiotherapy for brain metastases was approved by the radiotherapy physician. Varian’s EDGE (edge cancer treatment) was used, and volume-modulated radiotherapy was performed at a prescribed dose of 12 Gy three times. After radiotherapy, the symptoms of right limb weakness and numbness completely improved, but right limb weakness developed, which was accompanied by limb numbness and uncoordinated movement, 3 months later. Reexamination of brain MRI on March 23, 2020, showed a small, nodular abnormal signal in the left frontal parietal lobe, which was slightly larger than before, with a fuzzy edge. It was approximately 3.0 × 2.3 cm, and distinct patchy edematous foci were observed in surrounding areas. The patient was then given bevacizumab (7.5 mg/kg) to relieve the cerebral edema every 3 weeks. The patient’s symptoms persisted for another month and then became aggravated, and right limb dyskinesia was observed. The repeat cranial MR on May 11, 2020, showed that the left frontoparietal metastatic lesions rapidly increased to a size of approximately 4.5 × 2.5 cm, with significant surrounding edema. The patient still did not agree to surgical treatment and continued to be treated with bevacizumab. He died of intracranial hypertension more than 2 months later.

## Discussion

MFH is a type of soft tissue sarcoma originating from mesenchymal cells, accounting for 20% of all sarcomas, approximately one-fourth of which are radiation-related tumors with multidirectional differentiation abilities. First described by O’Brien and Stout (1) in 1964, MFH was originally named malignant fibrous xanthoma. In general, superficial MFH is mostly moderately malignant, while deep histiocytoma is mostly highly malignant. PMFHL is more common in males and in the right lung at older ages. Cancer cells are not easy to detect on sputum examination. Because the tumors grow outside the tracheal lumen, they are difficult to detect by fiberoptic bronchoscopy. The symptoms mainly include cough, blood in sputum, chest pain, dyspnea, fatigue, and weight loss. Rare symptoms include hypertrophic pulmonary osteoarthropathy, hypoglycemia, and neutrophilia, and some patients may be asymptomatic (4). Lymph node metastasis is rare in this disease, hematogenous metastasis is common, and the common metastatic sites are the brain and bone. In this case, chest X-ray showed that PMFHL manifested as a large noncavitary mass with a round shadow, uniform density, generally smooth edges, and inconspicuous lobulation. CT showed that the central density was low and the edges had increased density, and the surrounding shadow was irregular. Cystic degeneration and cavity formation, which are rare in MFH, were not observed.

The molecular mechanism of PMFHL occurrence and progression is still unclear. Some scholars have performed next-generation sequencing of tumor tissues, and the results showed that the *TSC2*, *ARID1B*, *CDK8*, *KDM5C*, and *CASP8* genes had mutations. Among them, the *TSC2* gene had the highest mutation frequency (15.64%), and an M280V missense mutation was found. Therefore, *TSC2* gene mutations are hypothesized to activate the mTOR pathway, resulting in abnormal cell growth and proliferation, which may be related to the occurrence and progression of this disease (5).

Diagnosing PMFHL is a multistep process. The most important step is to confirm that the lesions originate in the lungs. PET-CT can accurately exclude other parts of the body as the origin, especially the retroperitoneal region. Due to the low incidence of this disease, the small number of cases, and radiotherapy insensitivity, no standard chemotherapy regimen is currently available. Generally, MFH chemotherapy drugs are preferred. Commonly used drugs include ifosfamide and doxorubicin, which have low effective rates (6). Due to the presence of the *EML4-ALK* (E6:A20) rearrangement (fusion), which has not been reported in PMFHL, we can only explore this disease based on our experience in non-small cell lung cancer. A phase II study evaluated the efficacy of the PD-L1 inhibitor durvalumab for third-line treatment of advanced *EGFR*-mutated *ALK*-rearranged non-small cell lung cancer but reported no response in 15 non-small cell lung cancer patients with *ALK* fusion following durvalumab treatment (7). Alectinib is a second-generation *ALK*-positive lung cancer-targeting drug with a high penetration rate of the blood–brain barrier (63% to 94%). Therefore, the efficacy of alectinib in the treatment of brain metastases from lung cancer is markedly better than that of crizotinib. For patients with brain metastases from lung cancer, the median progression-free survival period after alectinib treatment is 27.7 months, while that of crizotinib is only 7.4 months (8).

Considering that traditional chemotherapy regimens and immunotherapy drugs were not very effective, we comprehensively considered the choice of alectinib and indeed achieved good efficacy, with an overall survival time of 19 months. The cause of death of the patient was intracranial hypertension caused by progression of the intracranial lesions and compression edema. Because PMFHL was less sensitive to radiotherapy and the diameter of the left parietal lobe metastasis was >2 cm, internal manifestations of complete liquefaction and necrosis were found, with few solid components. Surgical resection was the first choice of treatment. Although the X-Knife at 12 Gy was used for three segmentations, the symptom relief duration after radiotherapy was less than 4 months. Even when we used alectinib in combination with bevacizumab, there was no significant improvement in brain edema and tumor shrinkage, resulting in improved prognosis. The patient should have achieved better overall survival, but the patient refused surgical removal of the intracranial metastases. At the same time, PD-L1 expression was high, and the TPS was 80%. It was very regretful that immune checkpoint inhibitors were not used. Because tumor progression was due to oligoprogression of a single intracranial lesion, and local therapy should have been performed, including surgery and radiotherapy. If subsequent systemic progression occurs, then immunotherapy should be considered. Pembrolizumab application in PMFHL can achieve good outcomes (9).

## Conclusions

PMFHL is characterized by high malignancy, high recurrence and metastasis rates, a poor prognosis, and poor sensitivity to radiotherapy and chemotherapy. We recommend routine genetic testing of patients with PMFHL. Selecting targeted drugs with high sensitivity for possible mutation sites can often achieve better effective rates and longer survival times. Simultaneously, local treatment methods, including surgery and radiotherapy, are very important treatments when the disease progresses less. We first applied alectinib to a PMFHL patient with advanced EML4-ALK gene rearrangement (fusion) accompanied by brain metastasis. The treatment was effective. These findings need to be supported by further studies.

## Data availability statement

The original contributions presented in the study are included in the article/supplementary material. Further inquiries can be directed to the corresponding author.

## Author contributions

SZ collected, sorted, and analyzed the data and drafted the manuscript. XL collected and sorted the data. JC reviewed and revised the manuscript. All authors contributed to the article and approved the submitted version.

## Conflict of interest

The authors declare that the research was conducted in the absence of any commercial or financial relationships that could be construed as a potential conflict of interest.

## Publisher’s note

All claims expressed in this article are solely those of the authors and do not necessarily represent those of their affiliated organizations, or those of the publisher, the editors and the reviewers. Any product that may be evaluated in this article, or claim that may be made by its manufacturer, is not guaranteed or endorsed by the publisher.

## Abbreviations

PMFHL, Primary malignant fibrous histiocytoma of the lung; MFH, Malignant fibrous histiocytoma; CT, computed tomography; PET/CT, positron-emission tomography; FDG, ^18^F-fluorodeoxyglucose; TMB, tumor mutation burden; PD-L1, programmed death-ligand 1; TPS, tumor proportion score; MR, magnetic resonance.
